# Electrostatically Doped Junctionless Graphene Nanoribbon Tunnel Field-Effect Transistor for High-Performance Gas Sensing Applications: Leveraging Doping Gates for Multi-Gas Detection

**DOI:** 10.3390/nano14020220

**Published:** 2024-01-19

**Authors:** Khalil Tamersit, Abdellah Kouzou, José Rodriguez, Mohamed Abdelrahem

**Affiliations:** 1 National School of Nanoscience and Nanotechnology, Sidi Abdellah Technological Hub, Algiers 16000, Algeria; 2 Department of Electronics and Telecommunications, Université 8 Mai 1945 Guelma, Guelma 24000, Algeria; 3 Laboratory of Inverse Problems, Modeling, Information and Systems (PIMIS), Université 8 Mai 1945 Guelma, Guelma 24000, Algeria; 4 Applied Automation and Industrial Diagnosis Laboratory (LAADI), Faculty of Science and Technology, Djelfa University, Djelfa 17000, Algeria; kouzouabdellah@ieee.org; 5 Electrical and Electronics Engineering Department, Nisantasi University, Istanbul 34398, Turkey; 6 High-Power Converter Systems (HLU), Technical University of Munich (TUM), 80333 Munich, Germany; 7 Center for Energy Transition, Universidad Andres Bello, Santiago 8370146, Chile; jose.rodriguezp@uss.cl; 8 Electrical Engineering Department, Faculty of Engineering, Assiut University, Assiut 71516, Egypt

**Keywords:** Graphene nanoribbon (GNR), tunnel field-effect transistors (TFETs), junctionless (JL), quantum simulation, band-to-band tunneling (BTBT), work function (WF), gas sensors, electrostatics, nanoscale

## Abstract

In this paper, a new junctionless graphene nanoribbon tunnel field-effect transistor (JLGNR TFET) is proposed as a multi-gas nanosensor. The nanosensor has been computationally assessed using a quantum simulation based on the self-consistent solutions of the mode space non-equilibrium Green’s function (NEGF) formalism coupled with the Poisson’s equation considering ballistic transport conditions. The proposed multi-gas nanosensor is endowed with two top gates ensuring both reservoirs’ doping and multi-gas sensing. The investigations have included the I_DS_-V_GS_ transfer characteristics, the gas-induced electrostatic modulations, subthreshold swing, and sensitivity. The order of change in drain current has been considered as a sensitivity metric. The underlying physics of the proposed JLGNR TFET-based multi-gas nanosensor has also been studied through the analysis of the band diagrams behavior and the energy-position-resolved current spectrum. It has been found that the gas-induced work function modulation of the source (drain) gate affects the n-type (p-type) conduction branch by modulating the band-to-band tunneling (BTBT) while the p-type (n-type) conduction branch still unaffected forming a kind of high selectivity from operating regime point of view. The high sensitivity has been recorded in subthermionic subthreshold swing (SS < 60 mV/dec) regime considering small gas-induced gate work function modulation. In addition, advanced simulations have been performed for the detection of two different types of gases separately and simultaneously, where high-performance has been recorded in terms of sensitivity, selectivity, and electrical behavior. The proposed detection approach, which is viable, innovative, simple, and efficient, can be applied using other types of junctionless tunneling field-effect transistors with emerging channel nanomaterials such as the transition metal dichalcogenides materials. The proposed JLGNRTFET-based multi-gas nanosensor is not limited to two specific gases but can also detect other gases by employing appropriate gate materials in terms of selectivity.

## 1. Introduction

In today’s world, gas detection has become increasingly vital across a broad spectrum of applications, including biomedicine, security, environmental monitoring, and the pharmaceutical industry. Particularly noteworthy is the measurement and monitoring of gas emissions arising from micro and nanosystems (e.g., synthesis and processing of nanoparticles and nanomaterials, fabrication and operation of nanoelectronic devices, chemical reactions in medical nanodevices, etc.). This aspect is of paramount importance, necessitating the advancement of miniature gas sensors, namely the micro- and nano-sensors [1,2,3,4,5]. Among the most intriguing micro- and nano-sensors for gases, field-effect transistors (FETs) stand out. In practical terms, field-effect transistors can serve as gas sensors when one of their components is sensitive to a specific gas and is utilized as a sensing element. In the literature, various studies have explored nanoscale field-effect transistors with sensitive source and drain electrodes, some with the bare channel itself acting as the sensing element, and others with a sensitive metal gate. Note that the use of the bare channel as a gas sensing element can lead to the overlap of several mechanisms and phenomena, which complicates the thorough understanding of the behavior of FET-based gas sensors [6,7,8,9,10]. Particularly, gas-induced gate work function (WF) modulation in FETs-based gas sensors has proven to be an effective, non-destructive, complementary metal-oxide-semiconductor (CMOS)-compatible, and high-performance approach for gas sensing applications [11,12,13,14,15]. Its adaptability across a range of field-effect transistor sizes, from micro-scale to nanoscale, makes it versatile and attractive [16,17,18,19,20,21,22]. In fact, enhancing the performance of FET-based gas sensors with sensitive gates can be achieved through two primary approaches. The first approach involves developing new sensing gate based on high-selective and ultra-sensitive materials to gas measurands [11,12,13,14,23,24,25]. In literature, many sensing materials have been proposed and investigated in the framework of the gas-induced gate work function modulation, among other we cite the metals such as palladium (Pd) and platinum (Pt), and the organic conducting polymers, which can be employed to sense specific target gas molecules selectively, such as polyaniline (PANI) and poly-pyrrole-tetrafluoroborate (PPTFB) for ammonia (NH_3_) and CH_3_OH (MeOH) detection, respectively [26]. The second approach focuses on developing new transduction mechanisms to improve sensing figures of merit, we cite among others the use of new sensing regimes (e.g., band-to-band tunneling regime in carbon nanotube/ribbons FETs [27,28,29]), emerging field-effect transistors (e.g., tunnel field-effect transistors [30,31,32]), and electrostatic control-based improvement approaches (e.g., the dual gate configuration approach [33]), etc. In this context, tunneling field-effect transistors (TFETs) have been extensively investigated while exploiting the subthermionic subthreshold swing (SS < 60 mVdec) that can exhibit [26,30,31,32,34,35,36]. This approach can be understood by the fact that the subthreshold swing of field-effect transistors can be defined by the required gate voltage that can change the drain current by about one order of magnitude [37,38], and since the gas-induced gate work function modulation can be considered as an implicit amount of gate voltage [23], field-effect transistors with steep slope characteristics are highly desired for an ultra-high sensitivity to gate work function modulations in terms of drain current change [26]. However, the elaboration of tunnel FETs devices require the realization of abrupt doping junctions [39,40], which is not easy-to-make, especially in ultrascaled regime [41]. In this context, some reported works have proposed the junctionless tunnel field-effect transistor as gas sensor while ensuring the TFET doping profile via the electrostatic doping techniques [30]. 

Actually, the ambipolar behavior in tunnel field-effect transistors is a well-known phenomenon in TFET transfer characteristics, and it is unsuitable for switching applications due to the possible record of high off-state current. Leveraging the ambipolar behavior for multi-gas sensing, utilizing junctionless fabrication and electrostatic engineering techniques for fabrication facility, and exploiting the TFET subthermionic subthreshold swing for ultra-high sensitivity form an innovative idea that can provide a new push to the field. In our work, we propose a highly sensitive and selective multi-gas nanosensor by leveraging the gas-induced gate WF modulation principle. We incorporate the subthermionic subthreshold swing in GNR TFETs to enhance drain-current sensitivity, along with the junctionless paradigm and electrostatic doping to avoid abrupt junctions required for TFET operation. We also make use of the ambipolar behavior in junctionless tunnel field-effect transistors and employ doping gates as gas sensing elements for multi-gas detection. The computational method used to simulate the suggested multi-gas nanosensor involves solving the mode-space non-equilibrium Green’s function in a self-consistent manner with a two-dimensional Poisson equation considering the ballistic limit while taking into consideration the gas-induced change in gate work function in the nanodevice electrostatics [42]. Our investigation covers I_DS_-V_GS_ transfer characteristics, sensitivity, selectivity via n-type and p-type conduction branches, band diagrams, energy-position-resolved current spectra, and multi-gas sensing. The proposed sensor demonstrates high-sensitivity in the subthreshold regime, capable of detecting two different gases separately or simultaneously.

The subsequent sections of this paper are organized as follows: Section 2 meticulously unfolds the nanosensor structure and its operational principles, concurrently offering a comprehensive elucidation of the proposed multiple gas sensing approach. In Section 3, we delve into a detailed exposition of the quantum mechanical approach employed for simulation and analysis, specifically grounded in the solutions of the NEGF-Poisson computational couple within the ballistic limit. Section 4 takes center stage by presenting and thoroughly discussing the numerical results, providing valuable insights into the underlying physics. The concluding section not only synthesizes our findings but also delivers conclusions and offers perspectives for future exploration. The manuscript is complemented by an appendix detailing the computational approach used, thereby providing a comprehensive closure to the overall computational work.

## 2. Nanosensor Structure and Multi-Gas Sensing Principle

**Figure 1:**
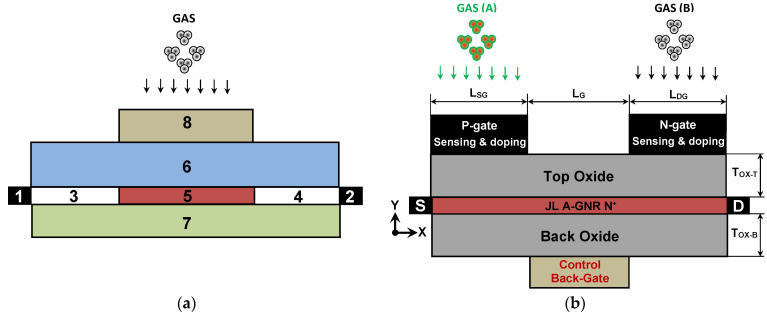
(**a**) The lengthwise cut view of the conventional structure of a work function-modulated FET-based gas sensor, commonly investigated in the literature. The elements 1 (2), 3 (4), 5, 6 (7), and 8 are source (drain) electrode, chemically or electrostatically doped source (drain) reservoir, channel, oxide (substrate/oxide), and control and sensing gate (e.g., single gate, double gate, gate all around, or multi-gate, etc.). (**b**) The lengthwise cut view of the proposed JL GNRTFET-based multi-gas nanosensor.

Figure 1a illustrates the 2D descriptive structure of the commonly investigated work function-modulated (T)FET-based gas sensor. The majority of reported WF-modulated FET-based gas sensors utilize a multi-functional main gate for both control and sensing, with various channel materials, configurations, and gate geometries [15]. It is noteworthy that, to the best of our knowledge, no WF modulated JL tunnel FET-based multi-gas nanosensor endowed with auxiliary gates for both doping and gas sensing, has been reported. Figure 1b shows the proposed multi-gas nanosensor, which is based on a junctionless graphene nanoribbon tunnel field-effect transistor (JL GNRTFET) endowed with source and drain doping gates acting as sensing elements. A heavily n-type doped junctionless armchair-edge GNR has been considered as channel material while avoiding the sharp abrupt junctions [43], which are disadvantageous in terms of facility of fabrication, reliability, and manufacturing cost. The required channel doping for tunnel field-effect transistor (TFET) operation (i.e., p-i-n or p-n [44,45]), has been ensured via an electrostatic doping using top source gate (at the left) and top drain gate (at the right) while applying the appropriate voltage. Note that these two gates act also as gas sensing elements, which distinguishes the proposed device in terms of multi-gas sensing. It is worth mentioning that the control gate has been taken to be placed at the bottom of the device (i.e., back gate configuration), in order to ensure the separation of the sensing face (gaseous environment) while avoiding any eventual gas-induced modulation. All gates are separated from the channel by top and back hafnium oxide (HfO_2_) layers, which are taken to have the same thickness (t_OX-T(B)_). The parameters L_G_ and L_S(D)G_ denote the control gate length and the length of source (drain) doping gate. Note that the S/D contacts have been appropriately considered to be ohmic since the AGNR is heavily n-type doped [46].

The working principle of the proposed sensor is based on the gas-induced modulation in metal gate work function [11,12,13,14,15,16,17,18,19,20,21,22,23,24,25,26]. Our proposed design can detect two different types of gases by recording a change in p-type (in the case of the first gas type) and n-type (in the case of the second gas type) conduction branches. Note that the suggested nanosensor can detect the gases separately or simultaneously. Equivalently, if it is about a unique gas-induced work function modulation at the level of source (drain) sensing gate, the induced electrostatic modulation affects the source (drain) band-to-band tunneling, thus the electron (hole) conduction branch will be modulated, while the hole (electron) conduction branch is still unaffected; and if two targeted gases are considered, the electron conduction branch is only affected by one of the two gases, at the same time, the hole conduction branch is affected by the other gas, which is highly efficient in terms of selectivity.

## 3. Quantum Simulation Approach

**Figure 2:**
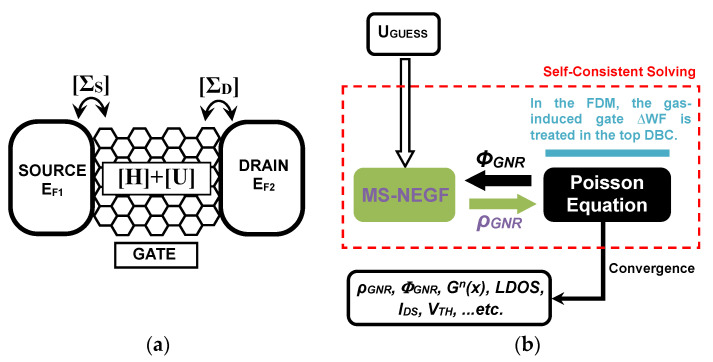
(**a**) The non-equilibrium Green’s function formalism for a generic nanoscale ballistic transistor. (**b**) The flowchart of the used quantum simulation approach.

Figure 2a illustrates the application of the non-equilibrium Green’s function formalism to a generic nanoscale transistor. Figure 2b shows the flowchart of the simulation used in the assessment of the proposed multi-gas nanosensor. This computational approach is based on the self-consistent solutions of the non-equilibrium Green’s function (NEGF) formalism coupled with the two-dimensional (2D) Poisson’s equation considering ballistic transport conditions [47,48,49,50]. The consideration of the 2D Poisson equation is based on the assumption that the potential in the width direction of the FET-based gas nanosensor remains constant. As shown in Figure 2b, starting from a potential profile guess, the mode space NEGF can be solved resulting a charge density output, which is considered as an input of Poisson’s solver. The latter computational bloc is approximately solved via the finite difference method (FDM) [33] while feeding back a potential profile to the NEGF solver. This coupled matrix calculation stops by a self-consistency in terms of the current and previous potential profile [42]. Then the nanodevice characteristics can be drawn from the Poisson’s solver and/or the NEGF solver, as shown in the same figure. It is worth mentioning that the mode space representation in the NEGF solver has been employed to avoid the computational burden associated with the real space-based quantum mechanical approach [42,47]. Note that a good agreement has been recorded between the two representations [47]. Inspecting the computational drawing of Figure 2b, we can see that the gas information is embedded in the concerned Dirichlet boundary condition (DBC), (i.e., at the top gates level) of the Poisson solver. It is worth noting that the potential at the top DBC has been fixed as *V = V_GS_ + Φ_S_ − (Φ_G_*_0_*+ ∆WF_G_)*, where *∆WF_G_=Φ_G−GAS_ − Φ_G_*_0_. Note that the parameters *V_GS_*, *Φ_S_*, *∆WF_G_*, and *Φ_G−GAS(G_*_0)_ denote the gate voltage, the work function of graphene nanoribbon, the gas-induced change in sensing gate work function, and the sensing gate work function after (before) the gas exposure, respectively [26]. The positive (negative) *∆WF_G_* means a gas-induced increase (decrease) in sensing gate work function [51]. The back DBC has been fixed normally without considering the gas-induced change in gate work function_._ For more details regarding the used simulation approach and the computational consideration of the gas-induced change in sensing gate work function, we refer to some important previous work including our computational proposal and numerical studies [23,26,33,51,52,53,54,55,56]. Note that the Appendix A provides the most important equations used in the quantum simulation. The simulator source code, along with all simulations, was implemented using Matlab environment software (version 2023), considering room temperature.

## 4. Results and Discussion

**Figure 3:**
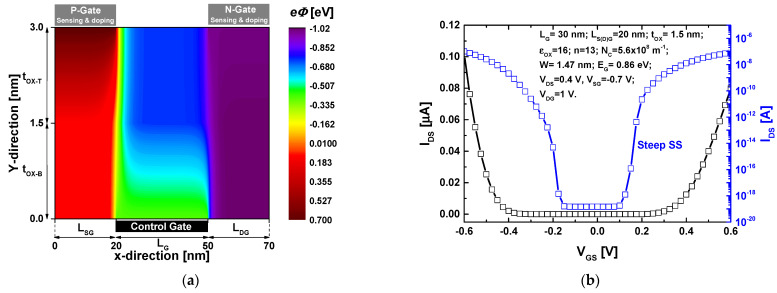
(**a**) The two-dimensional electron potential distribution of the proposed multi-gas nanosensor before gas exposure considering a V_DS_ = V_GS_ = 0.4 V as biasing conditions. (**b**) I_DS_-V_GS_ transfer characteristic of the proposed multi-gas nanosensor before gas exposure.

Figure 3a shows a colormap of the two-dimensional electron potential distribution drawn from the converged solutions of the Poisson’s solver considering a fresh nanosensor (before gas exposure). It is to Note that the adopted meshing space in the two directions has been taken to be equal to 1 Å [27]. We can clearly observe the electrostatic gating effects of the top doping gates ensuring the p-type and n-type electrical doping. As one can see the electrostatic gating of the back gate ensuring the control of the charge carrier within the channel. Figure 3b shows the I_DS_-V_GS_ transfer characteristic in linear and logarithmic scale of the fresh nanosensor. The nominal nanosensor parameters are indicated as inset. We can clearly see the ambipolar behavior of the transfer characteristics, which is an unsuitable feature in digital applications [57]. Note that many approaches (e.g., engineered chemical and electrical doping) have been proposed in literature to mitigate this detrimental effect [58,59,60,61,62]. It is important to mention that the two observed on-states are attributed to the band-to-band tunneling for electrons and holes, from the source to channel and from the drain to the channel, respectively [57]. In our proposed multi-gas sensor, the ambipolar behavior is exploited as asset in order to detect multiple gases. Inspecting the same figure, we can also see that the I_DS_-V_GS_ transfer characteristic enjoys with a subthermionic subthreshold swing (SS), (i.e., SS < 60 mV/dec [63]), of about 7 mV/dec, which is very beneficial for low power and high sensitivity applications [26]. It is worth mentioning that the steep swing factor has been found suitable for FET-based sensors considering measurand-induced change in effective gate voltage [26].

**Figure 4:**
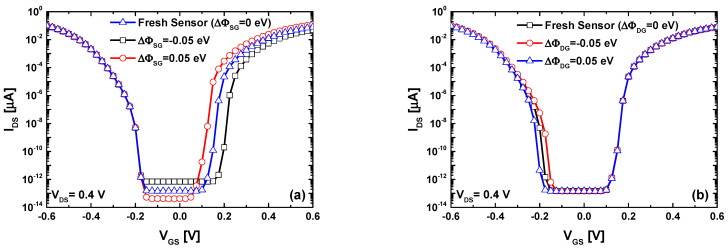
(**a**) The I_DS_-V_GS_ transfer characteristics of the proposed nanosensor considering the gas-induced change in the source sensing gate work function of ∆Φ_SG_ = −0.05 eV and 0.05 eV. (**b**) The I_DS_-V_GS_ transfer characteristics of the proposed nanosensor considering the gas-induced change in the drain sensing gate work function of ∆Φ_DG_ = −0.05 eV and 0.05 eV.

Figure 4a shows the behavior of the nanosensor in terms of the I_DS_-V_GS_ transfer characteristics considering two different values of gas-induced change in the source sensing gate work function (∆Φ_SG_). We can clearly see that the gas-induced positive (negative) ∆Φ_SG_ increases (decreases) the drain current (I_DS_) of the n-type conduction branch, while the p-type conduction branch is unaffected because the drain BTBT is not modulated. Figure 4b shows the I_DS_-V_GS_ behavior for the same values of the work function modulation considering a gas-induced change in the drain sensing gate work function (∆Φ_DG_). We can observe in this case that the drain current of the p-type conduction branch increases (decreases) due to the electrostatic gating effect of the negative (positive) ∆Φ_DG_, while the n-type conduction branch is unaffected since the BTBT at the source side is unaffected. This behavior has been previously observed using channel doping engineering as improvement approach targeting the mitigation of the ambipolarity in carbon nanotube TFETs and graphene nanoribbon TFETs for high-performance and low-power digital applications [57,64]. By comparing the current trends of the two cases, we can observe that the positive (negative) work function modulation affects the drain current conversely in the two cases, which can be considered as a clear selectivity. Thanks to the tunnel field-effect transistor working principle. Thereafter, we will explain in detail the observed modulation trends in drain current versus the gas-induced ∆Φ_DG_ and ∆Φ_SG_ using the energy-position-resolved current spectrum.

**Figure 5:**
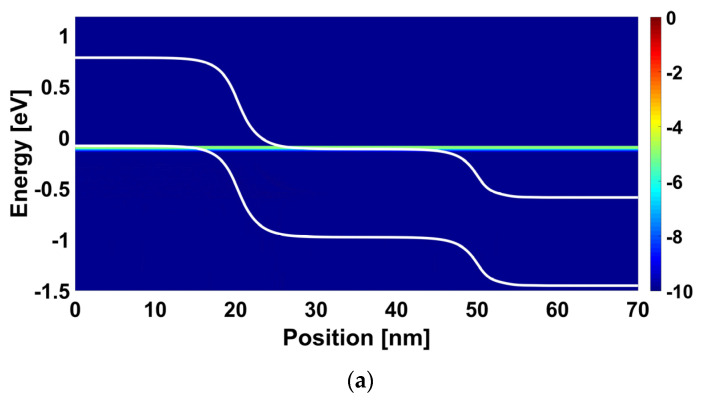

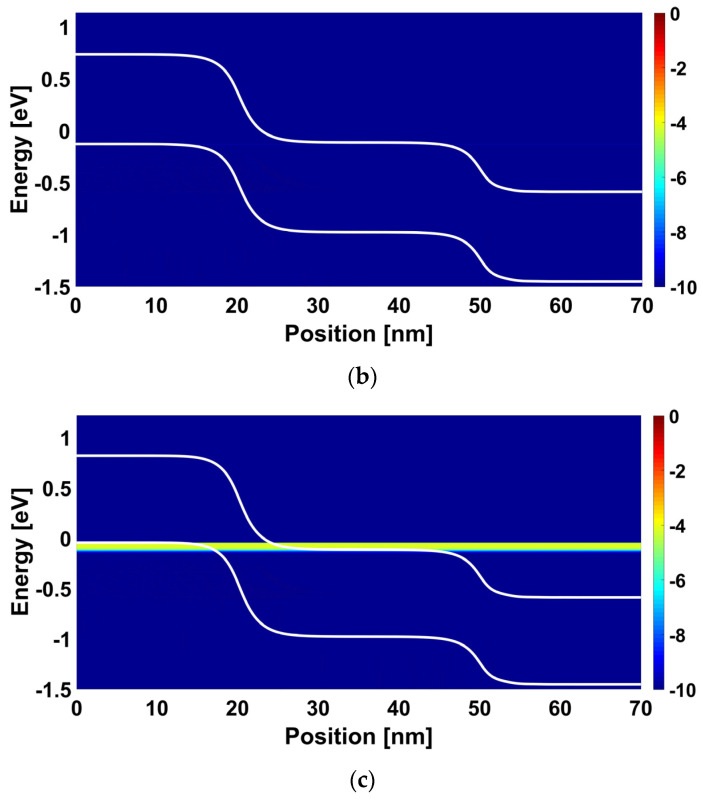
Energy-position-resolved current spectrum of the nanosensor with an active source sensing gate considering V_GS_ = 0.2 V and V_DS_ = 0.4 V for (**a**) Fresh nanosensor (before gas exposure, ∆Φ_SG_ = 0 eV), (**b**) ∆Φ_SG_ = −0.05 eV, and (**c**) ∆Φ_SG_ = 0.05 eV.

Figure 5 shows the energy-position-resolved current spectrum of the proposed multi-gas nanosensor with an active source sensing gate for fresh mode, positive and negative work function modulations, ∆Φ_SG_. We can see in Figure 5a a clear band-to-band tunneling current from source to the channel, which is attributed to the alignment of the valence band edge with the conduction band edge while allowing the tunneling of carrier. In Figure 5b, when considering a negative amount of gas-induced work function modulation of the source sensing gate, we can observe that the BTBT current is decreased due to the misalignment (i.e., lowering of the edge of the source valence band) leading to the pseudo turning-off while manifesting such current decreasing. However, Figure 5c shows that the consideration of a positive amount of gas-induced work function modulation of the source sensing gate increases the source BTBT due to the elevation of the edge of the source valence band leading to more injection of carrier into the channel. Note that these recorded behaviors in terms of energy-position-resolved current spectrum are in accordance with the I_DS_-V_GS_ behavior of Figure 4a.

**Figure 6:**
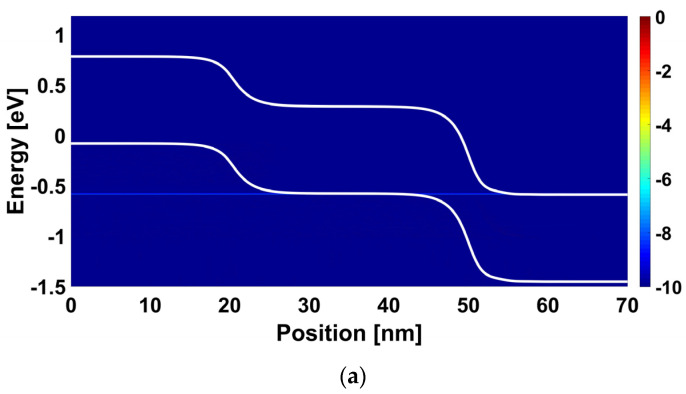

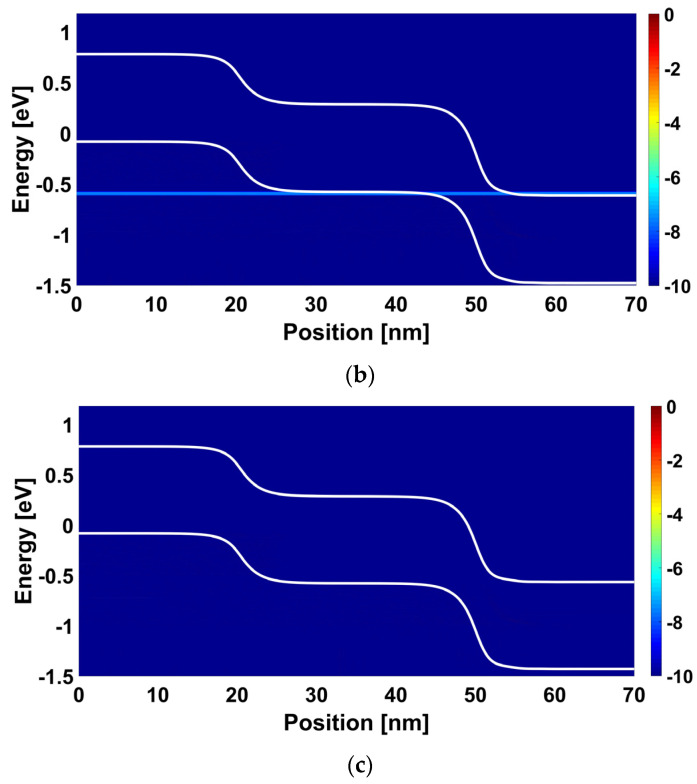
Energy-position-resolved current spectrum of the nanosensor with an active drain sensing gate considering V_GS_ = −0.2 V and V_DS_ = 0.4 V for (**a**) Fresh nanosensor (before gas exposure, ∆Φ_DG_ = 0 eV), (**b**) ∆Φ_DG_ = −0.05 eV, and (**c**) ∆Φ_DG_ = 0.05 eV.

Figure 6 shows the energy-position-resolved current spectrum of the proposed multi-gas nanosensor with an active drain sensing gate for fresh mode, positive and negative work function modulations, ∆Φ_DG_. We can see in Figure 6a that the BTBT near the drain is occurred due to the alignment of the valence band edge of the channel with the conduction band edge of the drain. By considering a negative (positive) work function modulation, we can observe that the aforementioned BTBT is increased (decreased) due the lowering (elevation) of the edge conduction band at the level of drain while boosting (braking) the BTBT, as shown in Figure 6b (Figure 6c).

**Figure 7:**
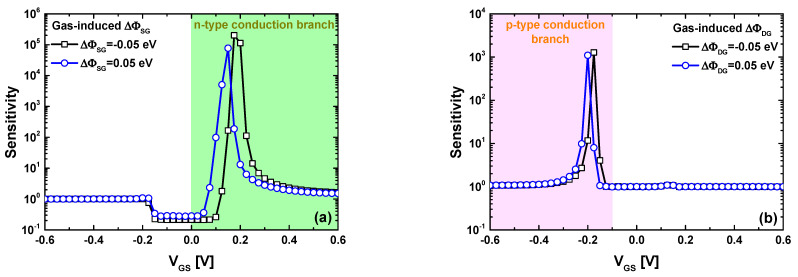
Sensitivity as a function of gate voltage for (**a**) the proposed nanosensor considering the gas-induced change in the source sensing gate work function and (**b**) considering the gas-induced change in the drain sensing gate work function of ∆Φ_D(S)G_ = −0.05 eV and 0.05 eV.

Figure 7 shows the sensitivity behavior of the proposed JLGNRTFET-based multi-gas nanosensor considering positive and negative values of gas-induced change in the source/drain sensing gate WF. Inspecting Figure 7a (Figure 7b), we can observe that the sensor manifests a high sensitivity in the steep slope regime (i.e., subthermionic SS) of the n-type (p-type) conduction branch. This behavior can be explained by the abrupt switching capability of the JLGNRTFET in presence/absence of BTBT juncture. Considering the obtained results, we can logically expect that operating the proposed JLGNR TFET-based multi-gas nanosensor (endowed with two different selective sensing gates) in dual gas environment allows to detect the concentration of the two gases selectively by tracking the drain current change in n-type (p-type) conduction branch for the first (second) gas monitoring, which will be investigated thereafter.

**Figure 8:**
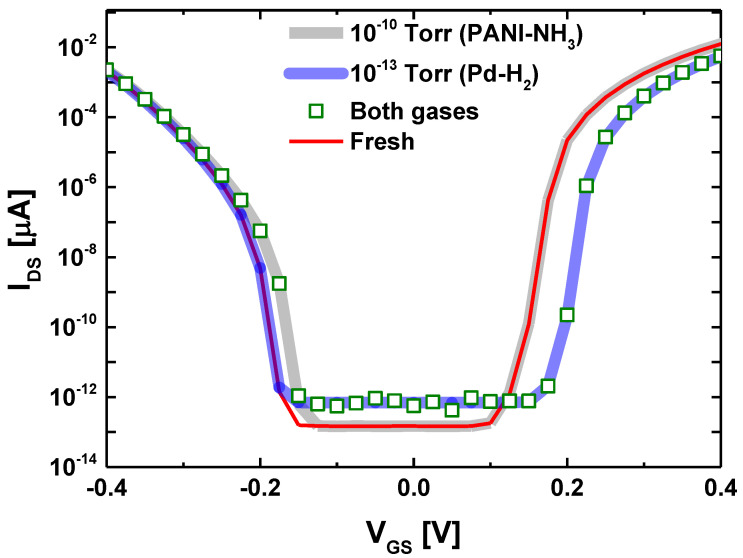
Simulation of the proposed JLGNRTFET-based multi-gas nanosensor in detecting two different type of gas.

In order to simulate the selectivity and sensitivity of the proposed JLGNRTFET-based multi-gas nanosensor in detecting two different gases, we have considered the palladium (Pd) as metallic source-sensing-gate, while we have considered the polyaniline (PANI) as polymer drain-sensing-gate to detect the hydrogen (H_2_) and ammonia (NH_3_) gases, respectively [26]. The simulated configuration is not limited to these two specific gases but can also detect other gases by employing appropriate gate materials in terms of selectivity. The gate metal work function variation is assumed to be predominantly attributed to exposure to specific gases (i.e., H_2_ and NH_3_). It is worth noting that background non-specific gases have also been considered in our computations, as detailed in [26]. The considered gas pressures have been taken to be 10^−10^ Torr of Ammonia while we have taken 10^−13^ Torr of Hydrogen. These two low gas pressures reflect the low work function modulations, which is practical in terms of performance projection and assessment. We can see in Figure 8 that the separate detection (i.e., separately gas sensing) of each gas can be performed by tracking the drain current in n-type or p-type conduction branch, depending on the targeted measurand. We can clearly see that the high sensitivity has been recorded in steep slope drain current region, or equivalently, where the sub-thermionic subthreshold swing is obtained, in both n-type and p-type conduction branch. Considering both gases, we can see that each gas only affects the concerned conduction branch without any modulation of the other conduction branch, which is a smart selectivity through the proposed sensor working principle and operating regime. It is worth noting that the proposed tunnel field-effect transistor is fully reconfigurable (electrostatically), where the gate biasing condition can be varied in order to get low subthreshold swing and ultra-high sensitivity in n-type and/or p-type conduction branch. From circuitry point of view, the adoption of advanced readout circuit that can track the drain current in n-type and p-type conduction branch is essential to well exploit the proposed multi-gas nanosensor. More importantly, the computational study and assessment of the effect of temperature variation on the sensing and transducing mechanisms are crucial and can serve as a subject for advanced investigations. It is worth noting that the temperature effect is beyond the scope of our current study. 

As a prospective avenue for further exploration, the utilization of carbon nanotube field-effect transistors [65,66,67,68] in conjunction with the proposed innovative approach calls for in-depth investigation. Furthermore, the integration of advanced bio-inspired algorithms, such as particle swarm optimization (PSO), genetic algorithms (GA), ant colony optimization (ACO), the fireworks algorithm (FWA), etc., and computational intelligence techniques like artificial neural networks (ANN), deep learning (DL), fuzzy logic (FL), etc. [69,70,71,72], into the simulator source code presents a compelling opportunity. This integration aims to optimize the multi-gas sensor’s structure, enhance its performance, and improve power efficiency. Additionally, considering that the gas-induced gate work function modulation can be perceived as an integral aspect of the gate voltage (i.e., effective gate voltage), exploiting the negative capacitance of ferroelectric nanomaterials [73,74,75,76,77,78,79] emerges as a promising strategy. This strategy has the potential to amplify the impact of gas-induced changes in gate work function on the device electrostatics and characteristics.

## 5. Conclusions

In conclusion, this paper introduced a novel junctionless graphene nanoribbon tunnel field-effect transistor (JLGNR TFET) as a versatile multi-gas nanosensor. The sensor was rigorously evaluated through quantum simulations, utilizing a coupled MS NEGF with 2D Poisson’s equation in the ballistic limit. The innovative design featured dual top gates, serving both source (drain) reservoir doping and multi-gas detection. The control gate has been placed separately (top and back) from doping and sensing gates to avoid any unintentional gas effect on the control gate while ensuring the nanosensor reliability. The study revealed that gas-induced modifications to the WF of the source and drain gates selectively influenced the device’s n-type and p-type conduction branches. The employed sensitivity was quantified by monitoring changes in drain current, with high sensitivity achieved in the subthermionic SS regime, particularly when exposed to minor gas-induced gate WF alterations. A thorough investigation of the underlying physics of the proposed multi-gas nanosensor has also been performed, utilizing band diagrams and energy-position-resolved current spectrum analyses. Extensive simulations were conducted, successfully demonstrating the device’s capability to detect two distinct gas types, both individually and simultaneously. The results highlighted the sensor’s outstanding performance in terms of sensitivity, selectivity, and electrical behavior. Importantly, this new multi-gas detection approach is not limited to graphene nanoribbon TFETs and can be adapted to various types of tunneling FETs based on traditional or emerging channel nanomaterials.

## Data Availability

The data that support the findings of this study are available from the first-corresponding author (K.T.) upon reasonable request.

## Appendix A

This appendix provides a brief overview of the NEGF-based quantum simulation utilized to assess the proposed JLGNRTFET-based multi-gas nanosensor. The used quantum simulation approach is well-established in the field of computational nanoelectronics, known for its accuracy, reliability, and ability to predict experimental outcomes. Moreover, it can comprehensively account for various physical phenomena, making it a potent atomistic simulation approach for proposing and evaluating new nanoscale transistors, including FET-based nanosensors [42,55].

The computation involves solving the Schrödinger equation through the mode space NEGF, coupled self-consistently with a two-dimensional Poisson equation under ballistic conditions while neglecting the scattering mechanisms [47,48]. In fact, the NEGF-based simulation allows for a rigorous treatment of quantum effects, electrostatics, and atomistic-scale features [42,55], which is not really within reach using the standard analytical models based on approximations. Note that this quantum approach has demonstrated a strong agreement with the experimental results of nanodevices based on carbon nanomaterials (i.e., carbon nanotube and graphene nanoribbon) [67,80,81]. Actually, the NEGF method necessitates the initial derivation of the retarded Green’s function, represented in the matrix form as follows [55]

(A1) $$G(E)={\left[(E+i\eta^{+})I-H_{PZ}-\sum_{S}-\sum_{D}\right]}^{-1}$$

Here, *E* represents energy, *η^+^* is an infinitesimally small number, *H_PZ_* denotes the Hamiltonian matrix for A-GNR based on the atomistic nearest neighbor p_Z_-orbital tight-binding (TB) approximation, *I* is the identity matrix, and Σ*_S(D)_* stands for the self-energy of the source (drain), respectively. It is noteworthy that, in the MS NEGF, only the first subband has been considered, ensuring both the necessary accuracy and simulation speed, which is an important trade-off in the NEGF-based numerical modeling. The computation of the self-energies and the retarded Green’s function make it possible to calculate the energy level broadening resulting from the source (drain) contact, denoted as *Γ_S(D)_*, as well as the local density of states for the source (drain), represented by *D_S(D)_* [47,48].

(A2) $$\Gamma_{S(D)}=i(\Sigma_{S(D)}-\Sigma_{S(D)}^{†})$$

and

*D*_*S(D)*_ = *G**Γ*_*S(D)*_*G*^*†*^
(A3)

The calculation of the charge density in armchair graphene nanoribbon channel can be routinely performed using the NEGF quantities mentioned above, as outlined in the following expression [47]

(A4) $$\begin{array}{l} Q(x)=(-q)\int_{-\infty }^{+\infty }dE\;\cdot \;\mathrm{sgn}\left[E-E_{N}(x)\right]\times \left\{D_{S}(E,x)\right.f(\mathrm{sgn}\left[E-E_{N}(x)\right](E-E_{FS}))\\ \;\;\;\;\;\;\;\;\;\;\;\;\;\;\;+D_{D}(E,x)f(\mathrm{sgn}\left[E-E_{N}(x)\right](E-E_{FD})\left.)\right\} \end{array}$$

In the given context, *q* represents the electron charge, *sgn* is the sign function, *E_N_* denotes the charge neutrality level, *f* is the Fermi function, and *E_FS(FD)_* corresponds to the Fermi level of the source (drain). Practically, the computation of mode space NEGF equations necessitates the determination of the on-site electrostatic potential. This can be derived from solving the two-dimensional Poisson equation given by:

Top of Form

(A5) $$\nabla^{2}U=\frac{-q}{\varepsilon }\rho$$

Here, *U* represents the electrostatic potential, *q* denotes the magnitude of the electron charge, *ε* stands for the dielectric constant, and *ρ* corresponds to the distribution of net charge density, encompassing the doping concentrations. After successfully solving the Poisson solver using the finite difference method and achieving computational convergence, or equivalently, self-consistency, as shown in the quantum simulation section above, the current can be computed through the following integral [33]

(A6) $$I=\frac{2q}{h}\int dE\;T(E)\left[f(E-E_{FS})-f(E-E_{FD})\right]$$

Here, *h* represents Planck’s constant, and *T(E)* denotes the transmission coefficient. The transmission coefficient can be determined by utilizing the issued NEGF parameters as

(A7) $$T(E)=Tr\;[\Gamma_{S}G\Gamma_{D}G^{†}]$$

Here, *Tr* signifies the trace operator. For a detailed understanding and view of the numerical approach employed in this computational study and investigation, we refer to key works in computational nanoelectronics [42,55,80,81,82,83,84,85].
